# Ultrasound-guided fine-needle aspiration for retrojugular lymph nodes in the neck

**DOI:** 10.1186/1477-7819-11-121

**Published:** 2013-05-30

**Authors:** Dong Wook Kim

**Affiliations:** 1 Department of Radiology, Busan Paik Hospital, Inje University College of Medicine, 633-165, Gaegeum-dong, Busanjin-gu, Busan, (614-734), South Korea

**Keywords:** Lymph node, Retrojugular, Fine-needle biopsy, Papillary thyroid carcinoma, Metastasis

## Abstract

**Background:**

No study on ultrasound-guided fine-needle aspiration (US-FNA) for the diagnosis of retrojugular lymph node has been reported. The present study aimed to introduce US-FNA techniques for retrojugular lymph node and to evaluate their efficacy.

**Methods:**

Of the 788 patients who underwent US-FNA of the cervical lymph node, 41 patients underwent US-FNAs of retrojugular lymph node and were included in this study. The adequacy and efficacy of US-FNA of retrojugular lymph node and related complications during or after the procedure were assessed.

**Results:**

Of the 41 patients, 35 (85.4%) were adequately diagnosed in cytological analysis; four predominantly cystic lymph nodes were identified. Based on cytohistopathology results, thyroglobulin measurement, tuberculosis polymerase chain reaction, and sonographic follow-up, malignant (n = 26) and benign (n = 15) lymph nodes were confirmed. When six lymph nodes with inadequate cytology were classified as benign and malignant, the sensitivity, specificity, positive and negative predictive values, and accuracy of US-FNA in differentiating malignant from benign lesions were 69.2% and 92.3%, 100% and 100%, 100% and 100%, 65.2% and 88.2%, and 80.5% and 95.1%, respectively. No substantial complications related to the US-FNA procedure were observed.

**Conclusions:**

The present US-FNA method may be helpful for the diagnosis of retrojugular lymph node.

## Background

Fine-needle aspiration (FNA) is widely accepted as an effective method for diagnosing nodular lesions in the head and neck [1-6]. The diagnostic accuracy of FNA for diagnosing lymph node metastasis in the neck ranges from 91% to 100% [7-9], and the most common primary malignancies involving the cervical lymph node are squamous cell carcinoma and papillary thyroid carcinoma [5]. Recently, analysis of the tumor marker concentration in the FNA washout has been found to improve the detection of lymph node metastasis in patients with known malignancies [10].

FNA is used worldwide as a first-line method for the diagnosis of palpable neck masses or lymph nodes, but ultrasound-guided FNA (US-FNA) can additionally be used to assess non-palpable or less palpable neck masses or lymph nodes. However, in some cases, US-FNA of neck mass or lymph node involves a difficult approach. In particular, US-FNA for neck masses or lymph nodes that are located behind major vessels is difficult, even for experienced practitioners. This procedure may be performed by directly puncturing the major vessel after compressing the major vessel with the US probe, but it carries a risk of inducing tumor seeding through the major vessel. To the best of my knowledge, no report has presented or recommended US-FNA sampling of a lymph node hidden by the internal jugular vein (called retrojugular lymph node).

The present study describes US-FNA techniques for retrojugular lymph nodes, and the adequacy and efficacy of US-FNA of retrojugular lymph node and related complications were assessed.

## Methods

### Patients

The study was approved by the Institutional Review Board (IRB 12-004). From January 2008 to December 2010, US-FNAs of cervical lymph nodes were performed in 788 patients (female: male = 565:223; mean age, 45.8 years; age range, 1 to 85 years) at our hospital. Among them, consecutive patients who underwent US-FNA of a retrojugular lymph node were selected for this study. Before US-FNA, all study patients were screened by a diagnostic neck US examination using a high-resolution ultrasound scanner (iU 22; Phillips Medical Systems, Bothell, WA, USA) with a 5- to 12-MHz linear probe. All US-FNAs were performed by a single radiologist with 8 years of experience.

The criteria for a patient to undergo US-FNA of a retrojugular lymph node included (1) the presence of a retrojugular lymph node with likely malignancy or tuberculous lymph node, as determined by diagnostic US; (2) the absence of an alternative, non-retrojugular lymph node with likely malignancy or tuberculous lymph node.

### Ultrasound-guided fine-needle aspiration of the retrojugular lymph node

**Figure 1:**
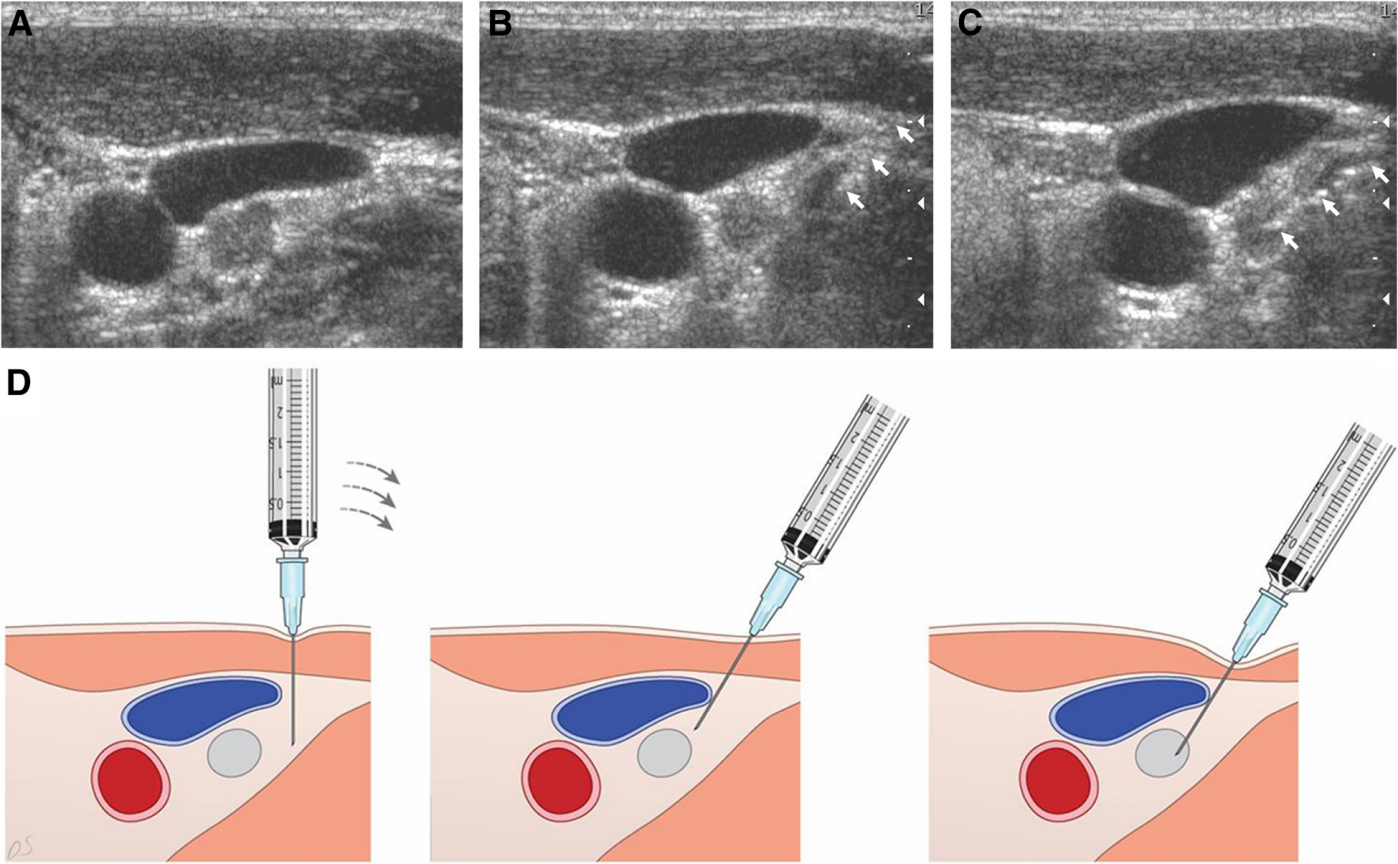
**A 48-year-old woman with a retrojugular lymph node in left side of neck on follow-up ultrasound (US) after total thyroidectomy for papillary thyroid carcinoma (4.1 × 5.3 × 5.5 mm).** The retrojugular lymph node was diagnosed as ‘lymph node metastasis from papillary thyroid carcinoma’ in cytology after US-FNA, and the patient underwent radioisotope therapy without neck surgery. **A.** Perpendicular puncture: a spot away from the lateral wall of the internal jugular vein. **B.** Lateral angulation of the syringe-needle unit (the echogenic line (arrows) indicates the needle shaft and tip). **C.** A successful puncture of the target after an appropriate approach of the syringe-needle unit (the echogenic line (arrows) indicates the needle shaft and tip). **D.** Drawings illustrating how to approach a lymph node located in the retrojugular area. Initially, the needle is perpendicularly inserted at the periphery of the internal jugular vein (left). The syringe-needle unit is then angled and the needle tip is wedged into the lesion (middle and right).

The assay was performed with a 10-ml plastic syringe attached to a conventional 23-gauge needle without an aspiration device. The patients were placed in the supine position and the exact position was decided according to the location of target node. For right retrojugular lymph node, the practitioner sat on the left side of the patient, facing the patient’s feet, because the practitioner was right-handed. For left retrojugular lymph node, the practitioner sat on the right side of the patient, facing the patient’s face. After the patient’s neck was sterilely prepared and draped, the target node was localized in the center of the US monitor using the US probe. During the entire procedure, the practitioner maneuvered the US probe in his left hand and the syringe-needle unit in his right hand. This positioning allowed the practitioner’s thumb to control the power of syringe aspiration by retracting the piston of the syringe. To prevent the direct puncture of major vessels, the practitioner introduced the tip of the needle away from the lateral wall of the internal jugular vein (Figure 1). Before needle puncture, it was helpful to visualize the motion of the tissue on the US monitor when the needle was gently moved back and forth on the skin surface. A shallow, perpendicular puncture was made several millimeters away from the lateral wall of internal jugular vein. After perpendicularly introducing the needle tip to the level of the posterior wall of the internal jugular vein, the practitioner laterally angulated the syringe-needle unit until the needle tip was facing toward the target. The target lesion was then kept centered in the transverse US image, and the practitioner advanced the needle into the target lymph node using an oblique puncture technique. With real-time monitoring on the US monitor, the needle tip could then be positioned within an appropriate area of the target. It was particularly important to thoroughly monitor the needle tip during the entire procedure. After the needle tip was placed in the appropriate position within the target, the sampling commenced using back-and-forth movements and a mixed sampling technique (that is, capillary sampling at first, followed by gradual aspiration). No local anesthesia was used in US-FNA for any patient.

Following US-FNA, each sample was immediately mounted onto a glass slide. In each US-FNA, the practitioner could obtain 4 to 6 slides by duplicating the smears from one needle pass and one-sampling. The slides were fixed in 95% ethanol and then sent for cytological analysis. For partially cystic lesions, the remaining aspirate within the syringe was sent for cell analysis, thyroglobulin titers, or tuberculosis polymerase chain reaction according to the clinico-sonographic indication. Electrochemiluminescent immunoassay using the Elecsys automatic system (Roche, Mannheim, Germany) was used for measurement of thyroglobulin titer from aspirates in US-FNA. Any patient complication that occurred during or after the US-FNA procedure was also recorded.

### Assessment of the final diagnosis for retrojugular lymph node

The assessment criteria for the final diagnosis of retrojugular lymph node included (1) histopathology results; (2) the presence of confirmative cytology findings, including typical papillary thyroid carcinoma or squamous cell carcinoma; (3) tuberculosis polymerase chain reaction results; (4) in aspirates, when the thyroglobulin titer of the retrojugular lymph node is >1,000 ng/ml or is 10-fold higher than the serum thyroglobulin level, it is classified as a metastatic lymph node from papillary thyroid carcinoma; and (5) when a retrojugular lymph node shows no malignant cytohistopathology, a negative thyroglobulin titer in aspirates, and no malignant finding in the initial and follow-up USs, it is classified as benign.

### Statistical analysis

The incidence of all retrojugular lymph nodes was statistically evaluated with regard to sex, age, and location using Fisher’s exact test. The adequacy of US-FNA for retrojugular lymph nodes was statistically evaluated depending on the largest diameter of the lymph node (≥10 mm and <10 mm) using Fisher’s exact test. The sensitivity, specificity, positive and negative predictive values, accuracy, and false positive and negative rates of US-FNA for the retrojugular lymph nodes were calculated by comparing the US-FNA results with the final results. For this analysis, lymph nodes showing inadequate cytology were classified as either benign or malignant. These data were evaluated using Fisher’s exact test. Values of *P* <0.05 were considered statistically significant. Data analyses were performed with SPSS for Windows (version 17.0.1; SPSS Inc., Chicago, IL, USA).

## Results

During the study period, 41 patients (female: male = 36:5; mean age, 44.2 years; age range, 22 to 65 years) underwent US-FNA of retrojugular lymph node (mean size, 9.2 mm; size range, 5 to 25.3 mm) because of a clinical or sonographic suspicion of malignant (n = 39) or tuberculous (n = 2) lymph node. Only one sampling was used for all US-FNAs, and 4 predominantly cystic lymph nodes were found in the diagnostic US. Retrojugular lymph node was located on the right (n = 23) and left (n = 18). There was no significant difference in the incidence of retrojugular lymph node according to age, sex, and location (*P* >0.05, Fisher’s exact test). Of the 41 patients, 35 (85.4%) showed adequate cytology in US-FNA of retrojugular lymph node. The diagnostic adequacy of US-FNA did not differ between retrojugular lymph nodes with the largest diameter ≥10 mm and those with the largest diameter <10 mm (*P* = 0.1336, Fisher’s exact test).

Of 39 suspicious malignant lymph nodes, histories of malignancy in the thyroid (n = 36), lung (n = 1), gastrointestinal tract (n = 1), and ovary (n = 1) were found. Based on the cytohistopathological results (n = 26), thyroglobulin titers (n = 5), tuberculosis polymerase chain reaction (n = 2), and long-term US follow-up (n = 8), 26 malignant and 15 benign lymph nodes were finally determined. The sonographic diagnoses, cytological analyses in US-FNA, and final diagnoses in the 41 retrojugular lymph nodes are summarized in Table 1. The retrojugular lymph nodes in ten patients were surgically removed and among them, six were thyroid cancers (five papillary thyroid carcinomas and one medullary thyroid carcinoma); one was lung cancer (squamous cell carcinoma); one was ovarian cancer (poorly differentiated carcinoma); and two were reactive hyperplasias. The non-surgical retrojugular lymph nodes in 31 patients included 17 thyroid cancers (papillary thyroid carcinoma) and 14 reactive hyperplasias, and 17 patients with thyroid cancer underwent only radioisotope therapy.

**Table 1:** **Sonographic diagnosis, cytological result in ultrasound-guided fine-needle aspiration** (**US-FNA), and final diagnosis in 41 retrojugular lymph nodes**

**Sonographic diagnosis (n)**	**Cytological result (n)**	**Final diagnosis (n)**
Indeterminate for malignancy (15)	Benign (13), suspicious for malignancy (2)	Metastasis from ovarian cancer (1), metastasis from PTC (2), RH (12)
Lymph node metastasis from thyroid malignancy (22)	Inadequate (6), benign (1), suspicious for malignancy (1), malignant (14)	Metastasis from PTC (21), metastasis for MTC (1)
Lymph node metastasis from non-thyroid malignancy (2)	Benign (1), suspicious for malignancy (1)	Metastasis from lung cancer (1), RH (1)
Tuberculous lymph node (2)	Benign (2)	RH (2)
Total (41)		

*MTC* medullary thyroid carcinoma, *PTC* papillary thyroid carcinoma, *RH* reactive hyperplasia.

Classifying six lymph nodes with inadequate cytology as benign and malignant, which included four predominantly cystic lymph nodes and two solid lymph nodes, the sensitivity, specificity, positive and negative predictive values, and accuracy of US-FNA in differentiating malignant from benign lesions were 69.2% and 92.3%, 100% and 100%, 100% and 100%, 65.2% and 88.2%, and 80.5% and 95.1%, respectively (Table 2). All 6 lymph nodes with inadequate cytology were confirmed as metastatic nodes from papillary thyroid carcinoma.

**Table 2:** Diagnostic index of ultrasound-guided fine-needle aspiration (US-FNA) for 41 retrojugular lymph nodes

**Items**	**Inclusion of inadequate cytology into benign category (%)**	**Inclusion of inadequate cytology into malignant category (%)**
Sensitivity	18/26(69.2)	24/26(92.3)
Specificity	15/15(100)	15/15(100)
PPV	18/18(100)	24/24(100)
NPV	15/23(65.2)	15/17(88.2)
Accuracy	33/41(80.5)	39/41(95.1)
False positive rate	0/15(0)	0/15(0)
False negative rate	8/26(30.8)	2/26(7.7)

*NPV* negative predictive value, *PPV* positive predictive value.

No substantial complications were observed for US-FNA of retrojugular lymph node, except for several cases of mild pain (5/41, 12.2%). The patients complaining of mild pain during or after the procedure did not take analgesics. No specific abnormality related to US-FNA was reported in the biopsy site on follow-up US over a 6-month period in any patient.

## Discussion

US-FNA for head and neck lesions is widely used because it is inexpensive, rapid, well accepted by patients and has a high diagnostic accuracy [5]. However, FNA for the initial diagnosis of primary malignant lymph node remains controversial because of its questionable accuracy for the diagnosis of nodal involvement of lymphoma or leukemia [8,11-13]. Nevertheless, US-FNA for the cervical lymph node is used as a first-line diagnostic tool because most malignant lymph nodes are metastatic rather than primary. In patients with a history of malignancy, FNA showed a high sensitivity because it only needs to evaluate whether malignant cells are present or not; the disadvantages of FNA include a high rate of nondiagnostic samples and false-negative results [14].

In the present study, the most common site of the primary malignancy was the thyroid (36/41, 87.8%), and the mean size of the retrojugular lymph nodes was 9.2 mm. The high incidence of retrojugular lymph node metastasis from primary papillary thyroid carcinoma cannot be explained at present. Despite the small size of retrojugular lymph nodes, US-FNA provided a good adequacy (35/41, 87.5%) and diagnostic accuracy (33/35, 94.3%). In addition, there was no significant effect of the node size (≥10 mm or <10 mm) on the adequacy of US-FNA for retrojugular lymph node. Because of the presence of malignant cystic lymph node (n = 4), the diagnostic accuracy of US-FNA for retrojugular lymph node was higher when six nodules showing inadequate cytology were considered to represent malignancy or excluded from the calculation than when they were considered to be benign.

The author did not prefer US-FNA for retrojugular lymph node as shown in the inclusion criteria. US-FNA for retrojugular lymph node was not performed when non-retrojugular lymph node could be substituted for retrojugular lymph node. In addition, the author has performed US-FNA for the cervical lymph node with the largest diameter of >5 mm. However, some practitioners may prefer the largest diameter of the cervical lymph node to be ≥10 mm. Regardless of the cutoff diameter, the author emphasizes that the most important factor for successful US-FNA of retrojugular lymph node is complete monitoring, to accurately determine the position of the needle tip under US guidance.

There are several limitations to the present study. First, of the 41 retrojugular lymph nodes, only 10 were surgically confirmed. Second, there was a high rate of primary thyroid malignancy (87.8%, 36/41), which may represent a bias. Third, of the 36 patients with a history of thyroid malignancy, only 9 (25%) underwent thyroglobulin measurement for FNA aspirates. Furthermore, tumor marker concentration in FNA washout was not analyzed. Finally, a single experienced radiologist performed US-FNA in all cases. For further clarification, a multicenter study may be required.

## Conclusions

The present study showed a good adequacy and accuracy in US-FNA of retrojugular lymph nodes and no substantial complications. Therefore, the present US-FNA method may be helpful for the diagnosis of retrojugular lymph nodes.

## Competing interests

No competing financial interests exist. The author alone is responsible for the content and writing of the paper. The author declares that he has no competing interests.
